# Hallermann–Streiff Syndrome and Lower Limb Lymphedema with Nasal Obstruction

**DOI:** 10.1155/2022/1520880

**Published:** 2022-12-06

**Authors:** Ana Carolina Pereira de Godoy, Henrique Jose Pereira de Godoy, Jose Maria Pereira de Godoy

**Affiliations:** ^1^Pediatric Intensive Care, Intensive Cardiac Surgery Pediatric-Hospital Da Criança e Maternidade-HCM, Medicine School of Sao Jose do Rio Preto (FAMERP), Sao Jose do Rio Preto, Brazil; ^2^Member Research Group, Clinica Godoy, Brazil; ^3^Department General Surgery, Medicine School in São José do Rio Preto-FAMERP, São José do Rio Preto, Brazil; ^4^Cardiovascular Surgery Department, Medicine School in Sao Jose do Rio Preto-FAMERP, São José do Rio Preto, Brazil; ^5^Undergraduate Medicine Course and the Stricto Sensu Postgraduate Course-FAMERP, CNPq (National Council for Research and Development), Brasília, Brazil

## Abstract

**Background:**

Hallermann–Streiff syndrome (HSS) is a rare congenital abnormality involving multiple craniofacial malformations, such as micrognathia, prominent frontal and nasal bones, vision defects, and dental anomalies, which can result in obstructive sleep apnea syndrome. The aim of the present study was to report a case of nasal obstruction in an individual with Hallermann–Streiff syndrome who had never breathed through the nose during treatment for lower limb lymphedema involving cervical lymphatic therapy. *Case Report*. An 18-year-old female adolescent with a diagnosis of HSS was sent from the genetics service of a teaching school for the treatment of lower limb lymphedema. At around 11 years of age, the patient began to present edema in the left leg, accompanied by broadening of the face and neck. The patient reported having obstructed nostrils and breathing through the mouth her entire life. On the second day of treatment, the patient reported being able to breathe through one of the nostrils, this had never occurred before. Based on this finding, the decision was made to include linear facial lymphatic drainage using the Godoy method, which led to the complete resolution of the nasal obstruction in the first 15 minutes of treatment. Nasal obstruction in children with Hallermann–Streiff syndrome may be caused by lymphedema.

**Conclusion:**

A specific lymphatic drainage technique, such as cervical lymphatic therapy and facial linear lymphatic therapy, can resolve the obstruction and maintain the nostrils unblocked for months.

## 1. Introduction

Hallermann–Streiff syndrome (HSS) is a rare congenital abnormality involving multiple craniofacial malformations, such as micrognathia, prominent frontal and nasal bones, vision defects, and dental anomalies, which can result in obstructive sleep apnea syndrome [1, 2]. When severe (apnea-hypopnea index: ≥30), noninvasive treatment options may be insufficient [2].

The genetic cause of this developmental disorder is unknown [3]. Individuals with HSS have clinical characteristics that overlap with some progeroid syndromes belonging to the category of laminopathies, such as Hutchinson–Gilford progeria syndrome and mandibuloacral dysplasia [3]. The condition is immediately recognized by a characteristic facial gestalt with a small face, prominent forehead, narrow nose, and micrognathia. Conventional cytogenetic studies have revealed normal chromosomes in most cases [4]. Hereditary dental conditions associated with disorders during the development of the teeth can cause shortened roots. Short roots constitute a rare developmental anomaly in permanent dentition, the etiology of which is not well established [5]. Craniofacial deformities, which are found in 98-99% of cases of HSS, are the main anomalies in affected individuals [6].

Lymphedema in individuals with HSS has not been described in the literature. The Godoy treatment method for lymphedema employs several therapeutic strategies, one of which is an intensive treatment, which enables reducing the volume of the edema by approximately 50% in five days [7].

The aim of the present study was to report the case with an important improvement of nasal obstruction in an individual with Hallermann–Streiff syndrome during treatment for lower limb lymphedema involving cervical lymphatic therapy.

## 2. Case Report

An 18-year-old female adolescent with a diagnosis of HSS was sent from the genetics service of a teaching school for the treatment of lower limb lymphedema. The mother reported that the patient was born with HSS, had been submitted to surgery for the treatment of aortic stenosis at four months of age, and had been in follow-up with physicians throughout the years of the evolution of the syndrome. At around 11 years of age, the patient began to present edema in the left leg, accompanied by broadening of the face and neck. The patient reported having obstructed nostrils and breathing through the mouth her entire life. She also had difficulty raising her arms and combing her hair.

**Figure 1:**
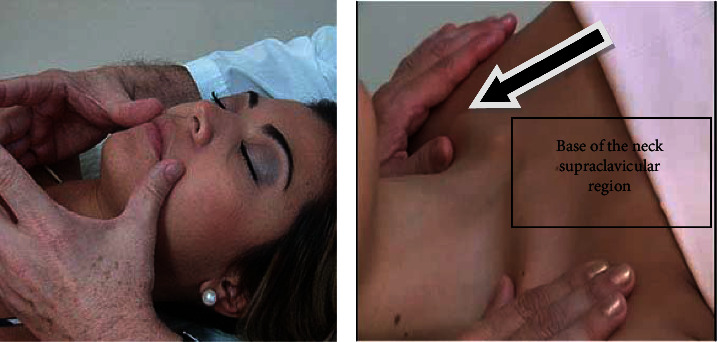
(a) Facial lymphatic drainage (linear movements) and (b) cervical lymphatic therapy Godoy method®.

The physical examination revealed clinical stage II lymphedema of the left lower limb beginning at the thigh, difficulty raising the arms and moving the neck, short neck, hardened region in the subcutaneous tissue of the face, and less hardened in the forehead region. The evaluation was made for lymphedema initially and after five days with multisegmented bioimpedance and volumetry by displacement water. Perimetric evaluation of the face was not performed, because the patient reported being able to breathe through one of the nostrils after cervical lymphatic therapy and linear facial lymphatic drainage, Figure 1, this had never occurred before.

Intensive treatment for lymphedema was proposed using the Godoy Method for five days [7]. Treatment consisted of eight hours/day of mechanical lymphatic therapy, 15 minutes/day of cervical lymphatic therapy, body, and facial manual lymphatic drainage, and the use of a laced, hand-crafted, nonelastic (grosgrain fabric) stocking as compression therapy.

On the second day of treatment, the patient reported being able to breathe through one of the nostrils, this had never occurred before. Based on this finding, the decision was made to include linear facial lymphatic drainage using the Godoy method®, which led to the complete resolution of the nasal obstruction in the first 15 minutes of treatment.

The mother reported that the patient's face had become thinner with treatment, which was confirmed by the comparison of photographs. The face had previously been thin and increased in volume after 11 years of age.

The patient has been in treatment for two years and her breathing has remained within the standards of normality for more than four months. When the obstruction recurs, a single session of cervical lymphatic therapy is sufficient to normalize her breathing and maintain it for months. An important improvement was also found with regard to motor aspects, as the patient was able to raise her arms and comb her hair, which was something she had not able been to do prior to treatment. This study received approval from the Human Research Ethics Committee of the São José do Rio Preto School of Medicine, SP, Brazil#5.165.749. The familiar responsible signed a consent form for publication.

## 3. Discussion

The present study describes an unexpected finding of the resolution of nasal obstruction in an adolescent with Hallermann–Streiff syndrome who had never previously breathed through the nose. The primary objective was to treat lower limb lymphedema, which has not previously been reported in this syndrome. The initial improvement in nasal breathing through one of the nostrils led us to include a specific nasal lymphatic drainage technique based on linear movements, which led to the resolution of the obstruction of both nostrils in approximately 10 minutes [8–10]. The most gratifying aspect was to see the patient's joy at being able to breathe through her nose. She concluded five days of intensive lymphedema treatment, which reduced the volume of the lower limb edema by 50%, and has had unobstructed nostrils for approximately two years.

The hypothesis for the broadening of the face and neck at 11 years of age is the lymphatic deficiency in the region that led to the development of lymphedema. The nasal obstruction since birth may have become aggravated over time. One of the routine techniques employed during intensive lymphedema treatment is cervical lymphatic therapy, which enables a reduction in facial fibrosis [8–16].

Breathing difficulty is a common finding in reports of HSS, suggesting the possibility of lymphedema. Therefore, the present case points to a novel treatment prospect that could alleviate this type of problem.

## 4. Conclusion

Nasal obstruction in children with Hallermann–Streiff syndrome may be caused by lymphedema. A specific lymphatic drainage technique, such as cervical lymphatic therapy and facial linear lymphatic therapy, can resolve the obstruction and maintain the nostrils unblocked for months.

## Data Availability

The data used to support the findings of this study are included within the article.
